# Plasmonic Nanoarray Biosensors for Non-Invasive Cancer Diagnostics

**DOI:** 10.3390/bios16080449

**Published:** 2026-08-18

**Authors:** Se Eun Kim, Hye Kyu Choi, Jin-Ha Choi

**Affiliations:** 1 School of Chemical Engineering, Jeonbuk National University, 567 Baekje-daero, Deokjin-gu, Jeonju-si 54896, Jeonbuk State, Republic of Korea; omija5911@gmail.com; 2 Department of Chemical and Biomolecular Engineering, Rutgers, The State University of New Jersey, Piscataway, NJ 08854, USA

**Keywords:** plasmonic nanoarray, non-invasive cancer diagnostics, liquid biopsy, urine, saliva, SERS, LSPR, metal-enhanced fluorescence, extracellular vesicles, early cancer detection

## Abstract

Early cancer detection can expand treatment options and improve patient survival, but it requires tests that can be repeated with minimal patient burden. Urine and saliva can be collected non-invasively and may contain cancer-associated nucleic acids, proteins, and extracellular vesicles. Clinical analysis of these body fluids is complicated by low biomarker abundance, inter-individual variation, and matrix components that interfere with surface-based sensing. Plasmonic nanoarray biosensors address some of these analytical constraints by concentrating local electromagnetic fields, supporting multiplexed optical readout, and accommodating surface chemistry and microfluidic handling. This review examines nanoarray architectures, fabrication methods, surface functionalization, and signal generation for cancer-associated biomarkers in urine and saliva. Localized surface plasmon resonance, surface-enhanced Raman scattering, and metal-enhanced fluorescence are discussed together with applications to bladder, prostate, pancreatic, oral, and head-and-neck cancers. Remaining barriers include biofouling, pre-analytical variation, fabrication reproducibility, limited validation using authentic biofluids, and incomplete sample-to-answer integration. Addressing these challenges through standardized biofluid processing, scalable nanoarray fabrication, and integrated microfluidic platforms will be essential for translating plasmonic nanoarray biosensors into clinically applicable cancer screening tools.

## 1. Introduction

Current cancer incidence and mortality estimates underscore the need for earlier detection. GLOBOCAN reported approximately 20.0 million new cases and 9.7 million deaths worldwide in 2022 and projected a substantial increase by 2050 [1]. In the United States, the American Cancer Society estimated 2,114,850 new diagnoses and 626,140 deaths for 2026 [2]. Tissue biopsy remains central to histological diagnosis and molecular profiling. However, sampling bias, spatial heterogeneity, limited repeatability, and procedural burden have motivated the development of complementary molecular tests [3,4]. The growing population of cancer survivors further increases the need for methods that can support repeated monitoring over time [5].

Conversely, liquid biopsy now contributes to genomic profiling, treatment selection, response monitoring, resistance detection, and minimal residual disease assessment [6,7]. ctDNA, extracellular vesicles, microRNAs, proteins, and circulating tumor cells can provide tumor-related information without surgical tissue removal [6]. Blood has the most established clinical role, but other body fluids, including urine, also contain cancer-related molecular signals; sensitivity, specificity, and standardization remain unresolved [8]. Urine and saliva permit repeated sampling with little procedural burden. Urine is particularly relevant to bladder cancer because tumor DNA and other molecular material can be released directly into the urinary tract [9]. Saliva is relevant to oral and head-and-neck cancers because it contacts the oral mucosa and contains measurable miRNAs and other analytes [10,11]. The present review focuses on plasmonic nanoarray strategies for detecting low-abundance cancer-associated signals in these accessible but chemically complex fluids [12].

Blood-based liquid biopsy currently has the strongest clinical evidence base among liquid-biopsy approaches, particularly for genotyping, therapy selection, resistance monitoring, and minimal residual disease assessment [6,7]. Blood, however, is a systemic compartment, and its molecular background includes cfDNA, proteins, vesicles, and inflammatory signals released from many non-tumor tissues [7,8]. Recent pan-cancer cfDNA methylome and fragmentome studies reinforce that early-detection performance depends on recovering weak tumor-derived signals from a much larger non-tumor background [13]. Sampling method also matters. Venipuncture is far less invasive than tissue biopsy, but it still requires trained personnel, supplies, and access to a clinic [8]. Urine and saliva, by contrast, permit repeated, low-burden sampling and may support longitudinal monitoring if downstream assays can tolerate pre-analytical and matrix variability [14,15]. This distinction is important for surveillance after treatment, risk stratification in high-risk groups, and decentralized testing workflows [12]. However, non-invasive collection does not remove the analytical challenges. Urine concentration varies with hydration, collection time, hematuria, renal handling, and normalization method [14,16]. Saliva composition varies with flow rate, circadian timing, collection conditions, oral hygiene, and other patient-specific factors [15]. Protease activity, inflammatory signals, and the oral microbial background can further complicate the sampled matrix [15,17,18]. A sensor built for these fluids therefore has to preserve target-specific signal generation in the presence of salts, proteins, enzymes, and nonspecific matrix background [14,15].

Urine is useful for practical reasons: it can be collected in relatively large volumes, without pain, and it has direct relevance to the urinary tract. Systematic work on urinary cancer biomarkers shows that collection timing, storage, centrifugation, and normalization conditions can strongly affect measured concentrations [14]. Creatinine or osmolality normalization can partially correct dilution, but hematuria and inflammation can still confound interpretation [16]. Thus, a urinary plasmonic assay should not be judged solely by buffer performance; it must also include matrix-matched calibration, background control, and robust sample handling. Saliva offers a different route for repeated, non-invasive sampling. Recent salivary biomarker reviews focus particularly on oral cancer and potentially malignant oral disorders, in which saliva collection is simple and the sampled fluid is anatomically close to the disease site [19,20]. In oral and head-and-neck cancer, salivary miRNAs and transcriptomic signatures have been investigated as disease-associated biomarkers [10,11,21]. Mucins, proteases, and other saliva components create risks of slower analyte transport, target degradation, and nonspecific retention at sensor surfaces; disease-associated salivary protease profiles further illustrate that enzymatic composition can vary across clinical conditions [15,18]. A saliva-facing optical assay therefore has to preserve the sampling advantage while reducing matrix-driven background signal.

Plasmonic nanoarrays are considered here as structured optical surfaces for urine- and saliva-based analysis. Metal nanostructures concentrate optical fields at metal-dielectric interfaces, allowing molecular binding near the surface to generate measurable optical changes [22,23,24]. Localized surface plasmon resonance (LSPR) detects local refractive-index changes [25,26,27], and arrayed nanoplasmonic structures have been used for label-free extracellular vesicle (EV) profiling [28]. Surface-enhanced Raman spectroscopy (SERS) and metal-enhanced fluorescence (MEF) are treated as distinct readout modes because their performance depends on reporter access, fluorophore-metal distance, and biofluid background. Here, nanoarray denotes a patterned sensing format with defined geometry, reproducible optical response, and spatially addressable capture regions. These characteristics are relevant when they improve capture chemistry, optical readout, or sample handling in urine- and saliva-facing workflows [23,28]. Nanoarray architecture is therefore evaluated as part of the diagnostic assay rather than as an isolated materials feature.

Several recent reviews have discussed optical nanomaterials, photonic sensors, and broader biosensor strategies for liquid biopsy [29,30,31,32]. These reviews provide broader context for the present focus on urine- and saliva-based sensing. In the following sections, matrix properties and analyte type are considered together with interface design, nanoarray architecture, readout, and validation.

This review links biofluid biology and biomarker class to nanoarray design, optical readout, and clinical application. The emphasis is on early detection, repeated sampling, matrix control, and interpretable results from urine- and saliva-based liquid biopsy [9,12]. The overall workflow is summarized schematically later in this review.

## 2. Non-Invasive Biofluids and Cancer Biomarkers

### 2.1. Urine, Saliva, and Other Biofluids

Urine and saliva are not simply less invasive versions of blood. They are different matrices, and each is useful only when the target cancer can release measurable material into that fluid. Urine is most relevant for urological cancers. Bladder tumors can shed tumor DNA and other molecular material directly into urine [9]. In prostate cancer, recent studies have evaluated urinary extracellular-vesicle miRNAs for non-invasive screening, diagnosis, and assessment of progression [33,34,35]. These examples explain why urine is often selected for urological applications: it can be collected in relatively large volumes, without venipuncture, and repeatedly over time. The limitation is that urine composition is variable. Hydration, hematuria, pH, osmolality, storage, urea, creatinine, and salts can affect both biomarker concentrations and the optical background [14,16].

Saliva is more directly linked to oral and head-and-neck cancers because it contacts the oral and oropharyngeal mucosa. Saliva-based oral cancer studies have examined nucleic acids, proteins, metabolites, microbial signals, and extracellular vesicles [10,19,36]. Salivary miRNAs and transcriptomic markers have also been evaluated [10,11,21]. At the same time, mucins, proteases, microbes, and food- or hygiene-related residues can affect target transport, stability, and surface background [15,18]. Assay design must therefore account for the matrix behavior of each biofluid. The major analytical characteristics of these biofluids are compared in Table 1.

### 2.2. Cancer Biomarkers in Non-Invasive Biofluids

Biomarker selection must match both cancer type and sample matrix. Cancer-associated changes in miRNA expression can be measured in extracellular miRNAs that remain protein-bound or enclosed within vesicles [37,38]. Their short length makes extraction, normalization, and sequence-specific recognition important sources of variation [39]. Urinary miRNAs are considered mainly in urological cancers, whereas salivary miRNAs are considered mainly in oral and head-and-neck cancers [10,11,40].

cfDNA and ctDNA provide information on mutations, methylation, copy number and fragmentation [6,7]. In early-stage disease, the tumor-derived fraction can be very small, which is why a low analytical LOD alone is not enough [13]. Urine is particularly relevant to bladder cancer because tumor DNA can be released into the urinary tract [9]. For nanoarray assays, cfDNA is most useful when the readout is tied to a defined mutation, methylation marker, or targeted panel rather than to total DNA accumulation.

EVs carry proteins, lipids, metabolites, DNA, mRNA, and miRNA from their cells of origin [41,42]. They have also been used as analyte carriers or targets in clustered regularly interspaced short palindromic repeat (CRISPR)-based diagnostic strategies [43]. Vesicle encapsulation can protect molecular cargo, but EV populations are heterogeneous. Assays that capture total EVs, tumor-enriched EVs, or marker-defined EV subpopulations measure different biological fractions [41,44]. Protein biomarkers remain relevant when they connect to established clinical workflows, as illustrated by multiplex PSA-related SERS testing [45].

### 2.3. Analytical Challenges in Non-Invasive Biofluids

The main analytical challenge is that early cancer signals are weak and the matrix background is strong. Plasma cfDNA studies show that tumor-derived fragments can be rare within a much larger non-tumor background [13,46], and single-EV liquid-biopsy work makes the same caution for blood explicit: tumor EVs can be rare within a much larger non-tumor vesicle background [47]. This blood-based evidence illustrates the general low-signal problem, but it is not direct evidence for urine or saliva. In urine, documented variation in salts, urea, creatinine, proteins, hematuria, and dilution creates conditions that can alter target recovery, bulk optical background, and nonspecific adsorption [14,16]. In saliva, mucins and proteases can slow transport or alter protein targets, while microbes and oral inflammation can add disease-unrelated background [15,18].

Performance reporting should reflect these matrix effects. A limit of detection (LOD) measured in buffer is useful for analytical comparison but does not establish performance in clinical urine or saliva. Studies using authentic specimens should report collection, processing, storage, normalization, recovery, blank-matrix response, baseline drift, chip-to-chip variation, assay failures, and agreement with a prespecified clinical endpoint [14,15]. EV assays should additionally define the EV source, isolation or capture method, characterization, and measured subpopulation [44]. Preconcentration, antifouling chemistry, CRISPR-assisted recognition, and multiplexed readout should be linked to the specific biofluid problem they are intended to solve.

**Figure 1:**
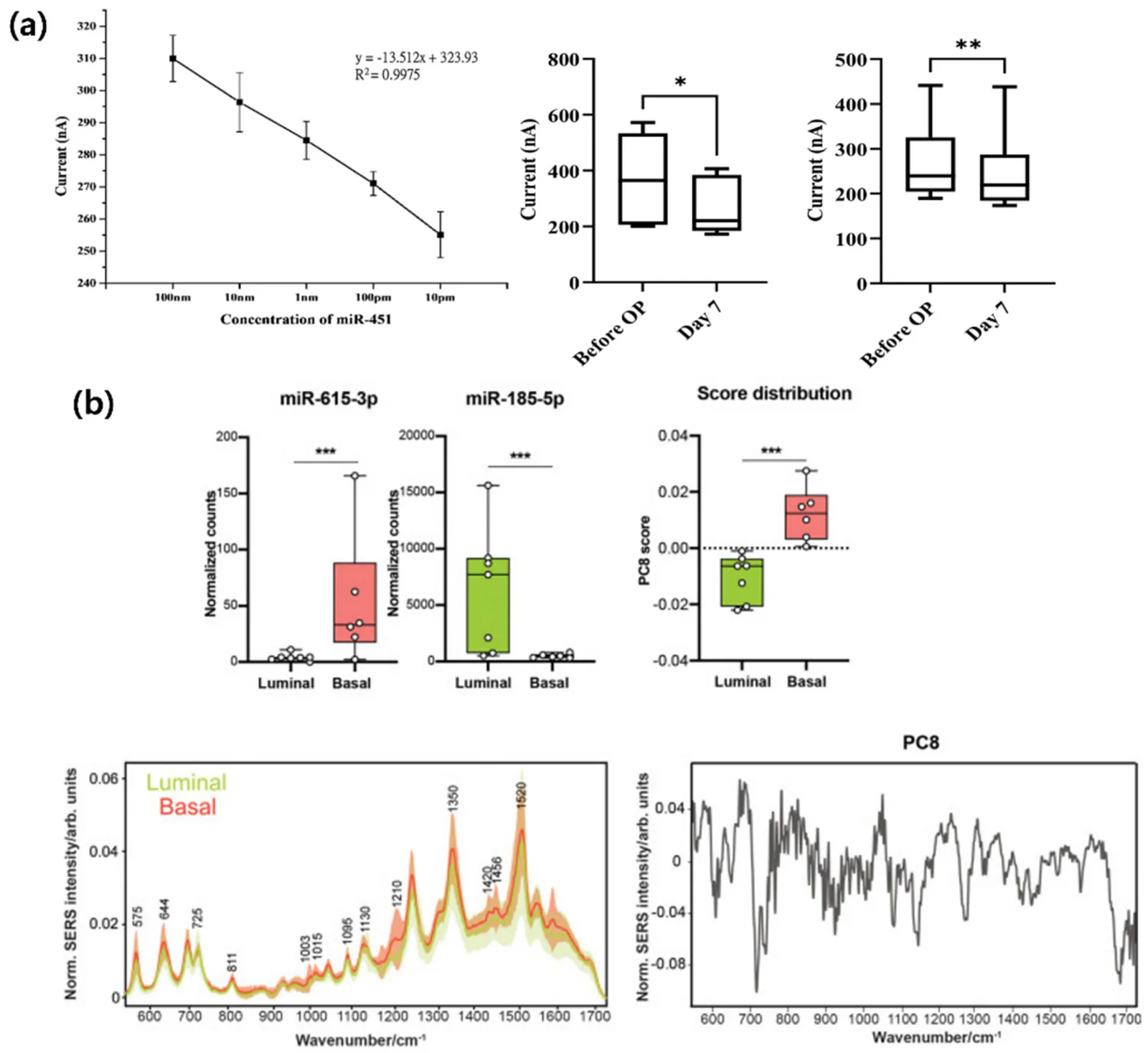
Selected urinary biomarker measurements. (**a**) Concentration-dependent electrochemical detection of urinary exomiR-451 and preoperative and 7-day postoperative measurements of exomiR-21 and exomiR-451. In the postoperative comparison, * denotes *p* = 0.0203 for exomiR-21 and ** denotes *p* = 0.0081 for exomiR-451. Adapted from Ref. [34]. © 2022 by the authors. (**b**) Urinary miRNA expression and SERS profiles for molecular stratification of luminal and basal bladder cancer. *** *p* < 0.001. Adapted from Ref. [48]. © The Author(s) 2022.

Chiou et al. demonstrated concentration-dependent electrochemical detection of urinary exomiR-451 and reported preoperative and 7-day postoperative measurements of exomiR-21 and exomiR-451 (Figure 1a) [34]. Moisoiu et al. compared urinary miRNA expression and SERS profiles between luminal and basal bladder cancer subtypes (Figure 1b) [48].

## 3. Plasmonic Nanoarray Platforms for Non-Invasive Biofluid Analysis

### 3.1. Nanoarray Architectures

Nanoarray architecture determines where the enhanced electromagnetic field sits, how large the sensing volume is, and how strongly the optical response changes after molecular binding [22,25]. For urine and saliva, this matters because target analytes arrive with salts, proteins, enzymes, mucins, microbial products, cells, and extracellular vesicles that are present during surface exposure and may compete for access to the sensing region [14,15]. A strong simulated hotspot is not enough. The architecture has to retain a measurable and reproducible signal after functionalization, matrix exposure, washing, and repeated readouts.

Nanohole arrays are well established for transmission-mode plasmonic sensing. Their resonance position and sensitivity depend on aperture geometry, periodicity, local refractive index, and analyte transport near the aperture [49,50]. The collinear geometry is compatible with compact readers because illumination and detection can share the same optical axis. Arrayed nanoplasmonic platforms have been used for label-free EV profiling [28], and recent single-EV assays have concurrently measured surface proteins and miRNAs, although not all such systems are plasmonic [51]. Electrokinetically enhanced EV capture and plasmon-enhanced single-EV fluorescence extend this EV-oriented approach [52,53].

Nanorod and nanoellipse arrays use anisotropic plasmon modes. Their longitudinal resonance depends strongly on aspect ratio and local dielectric environment, which enables spectral tuning [25]. A gold nanorod assay measured the oral-cancer-associated proteins CYFRA21-1 and CA125 in prepared mixtures and artificial saliva rather than patient saliva [54]. Porous gold nanorods have also been used for MEF-based multiplex miRNA detection [55]. These studies establish spectral-tuning and fluorescence-enhancement capability, but results obtained in buffer or artificial saliva require confirmation in clinical saliva, where fouling and transport differ [15].

Nanodisks, nanoparticle lattices, and surface-lattice-resonance arrays offer a different balance. Their advantage lies less in maximum hotspot intensity than in producing more uniform optical responses across a larger chip area. Surface lattice resonances can narrow spectral features by coupling localized plasmon modes to diffractive lattice modes, a design principle that has been reviewed extensively in the Surface Lattice Resonances (SLR) literature [56]. Centimeter-scale nanoparticle arrays featuring ultranarrow SLRs exhibit large-area fabrication and consistent optical properties [57]. Although these qualities are necessary, they do not alone confirm the ability to transfer calibration across different positions and manufacturing batches.

Self-assembled or colloidal-mask structures are attractive when low-cost, large-area fabrication is more important than nanometer-perfect geometry [25,58]. These methods can generate triangular, disk-like, or lattice-like plasmonic features without serial writing, but they also introduce packing defects and local variability. Particle-size distributions, vacancy defects, and imperfect packing can broaden resonance features and create spatial heterogeneity. In complex biofluids, this local variability may become especially consequential because a limited subset of field-sensitive regions can dominate the measured response. The performance metric should therefore be the distribution of the signal across many sensing regions rather than the best spectrum from a single selected location.

Optical metasurfaces and nanophotonic resonant surfaces broaden the design space beyond conventional metal arrays. Recent metasurface reviews emphasize that plasmonic, dielectric, and hybrid resonances can be engineered for biosensing and imaging [59]. A 2025 review on nanophotonic EV sensing specifically compares dielectric and plasmonic metasurfaces for label-free protein and EV detection, highlighting the relationship among resonance quality factors, evanescent-field overlap, and analyte size [60]. For urine and saliva diagnostics, this implies that the optimal structure depends on whether the target is a short nucleic acid, a protein, or a vesicle hundreds of nanometers wide.

Architecture should be selected according to analyte size and matrix composition. Short-miRNA assays require recognition designs that keep hybridization or reporter generation within the plasmonic near field [25,61]. Protein assays require receptor orientation and antifouling layers that preserve binding without moving the event too far from the metal surface; current evidence for this trade-off is largely drawn from adjacent electrochemical or porous-silicon systems [62,63,64]. EV assays require capture regions accessible to larger vesicles and a clear definition of the measured EV population [42,44]. No single geometry is optimal for all three analyte classes.

Initial sample contact is also architecture-dependent. An EV-oriented plasmonic array or nanowell platform [28,52] would require an added nucleic-acid recognition or amplification module before use for short miRNAs; this is a design inference rather than a demonstrated application. A nanorod MEF platform loses signal gain when fluorophores lie outside the enhancement zone because enhancement and quenching are distance-dependent [65,66]. High-Q lattice resonances can resolve small refractive-index shifts [56,57], but calibration transfer must still be demonstrated across chip positions and fabrication lots.

Architecture is therefore part of the assay workflow, not an isolated optical design problem. In urine, hematuria and abundant urinary proteins can alter bulk optical background and surface accessibility before target capture [14,16]. In saliva, mucins and microbial products may adsorb to sensing surfaces and reduce the effective number of available binding sites; this is a matrix-driven design concern inferred from the documented composition and variability of saliva [15,19]. A clinically useful nanoarray should be reported with both optical and matrix metrics: resonance position, linewidth, spatial uniformity, nonspecific adsorption, recovery after spiking, and signal drift after washing. Designs intended for miRNAs, proteins, and EVs may therefore require different capture densities, spacer lengths, washing steps, and readout windows even when the same plasmonic material is used.

The architectures therefore address different analytical constraints. Nanoholes support transmission-mode refractive-index sensing and near-aperture trapping [49,50]; nanoplasmonic arrays and nanowells have been developed for EV-oriented measurements [28,52]. Nanorods provide spectral tuning and MEF [25,55], whereas nanodisks and surface-lattice-resonance arrays favor large-area optical uniformity and narrow resonances [56,57]. Self-assembled arrays offer lower-cost fabrication at the expense of greater structural variation [58]. Their relevance to non-invasive cancer diagnostics depends on performance after exposure to the intended biofluid.

For early-detection use, the preferred architecture is the one that preserves diagnostic contrast in authentic urine or saliva, not the one that gives the strongest simulated field in a clean medium [14,15]. This is also why array-to-array reproducibility and spatial uniformity matter; repeated sampling is only useful if the same patient sample would be interpreted similarly across chips and readers. For patient testing, this also means the same architecture should tolerate realistic washing, storage, and operator-to-operator variation without altering the interpretation of weak-positive samples. These requirements are especially important for longitudinal testing across changing collection conditions.

For urine- and saliva-based assays, selection begins with the matrix and analyte, followed by interface design, architecture, readout, and validation. Urine-facing devices should first accommodate dilution, ionic-strength and pH variation, hematuria, and urinary proteins, whereas saliva-facing devices must manage mucin-driven viscosity, proteolysis, and oral-microbial background [14,15,16,18]. The analyte then sets the spatial constraint: short nucleic acids require accessible probes within the plasmonic near field, proteins require receptor orientation and controlled passivation thickness, and EVs require capture regions that remain accessible to vesicles while defining the measured subpopulation [25,42,44,61]. Nanoarray geometry and optical mode should be selected only after these matrix and interface constraints are specified. Recent systematic LSPR nanoarray data further show that particle diameter, out-of-plane thickness, surface roughness, substrate, and surrounding refractive index can all change resonance position, linewidth, and refractive-index sensitivity [67]. This matrix-to-validation selection framework is summarized schematically later in the review.

**Figure 2:**
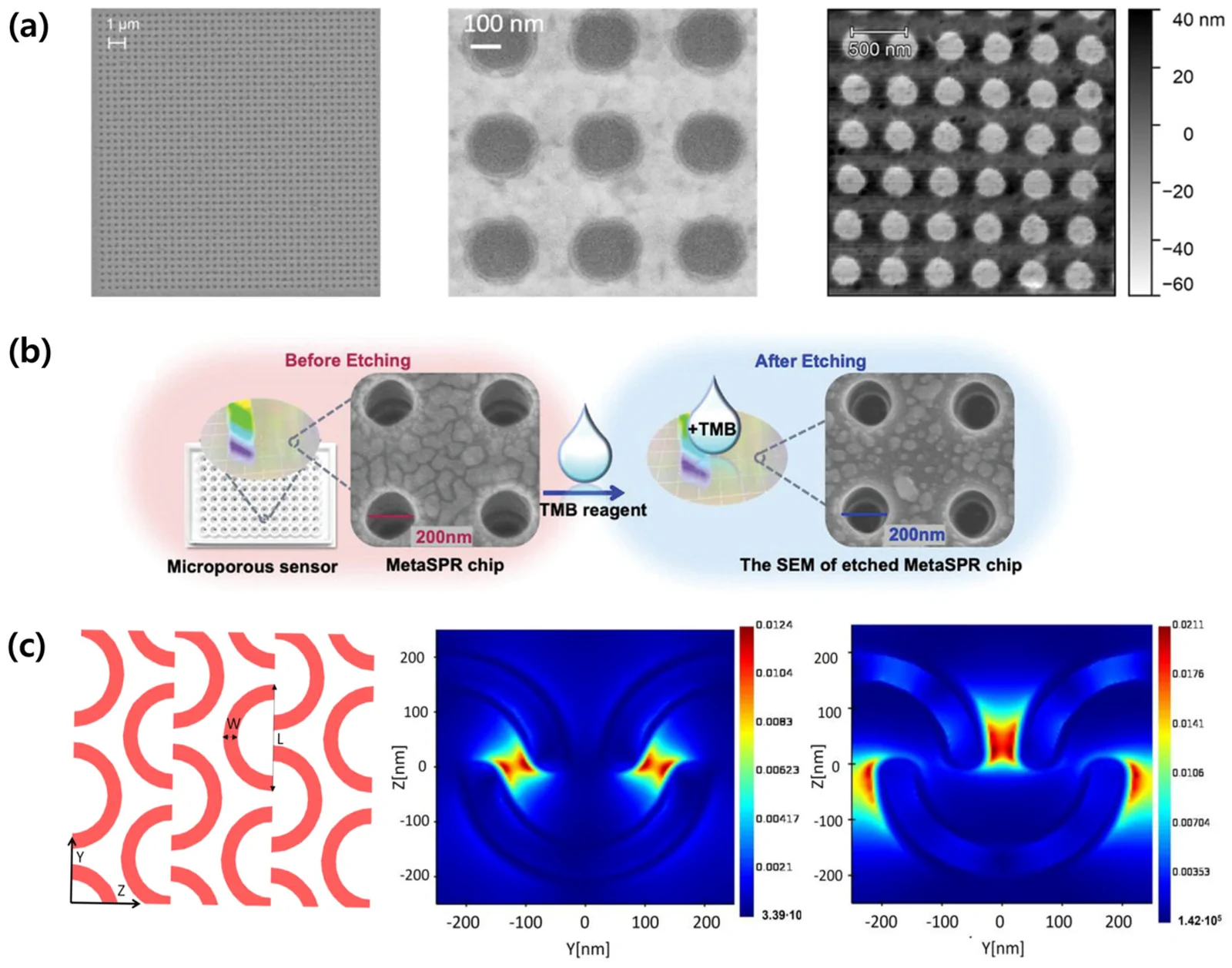
Structural characterization, etching-induced surface changes, and simulated electric-field distributions in plasmonic nanoarrays. (**a**) Structural characterization of the periodic nanocup array of the MetaSPR chip. Adapted from Ref. [68]. © 2023 The Authors. (**b**) Schematic and SEM images of the MetaSPR chip before and after oxTMB-mediated etching of the metal coating. Adapted from Ref. [68]. © 2023 The Authors. (**c**) C-shaped nanopattern design and simulated electric-field distributions at 800 and 1100 nm. Adapted from Ref. [69]. © 2023 by the authors.

Li et al. characterized the periodic nanocup architecture of the MetaSPR chip (Figure 2a) [68]. They further showed that oxTMB-mediated etching reduced the metal coverage on the chip surface (Figure 2b) [68]. Milenko et al. presented a C-shaped nanopattern and simulated its electric-field distributions at 800 and 1100 nm (Figure 2c) [69].

### 3.2. Fabrication and Surface Engineering

Fabrication controls whether the optical structure can be reproduced; surface engineering controls whether the right biomolecule reaches that structure. For urine and saliva, these two issues cannot be separated because matrix components present during exposure create a substantial surface-fouling risk [14,15]. A chip with excellent optical uniformity but weak antifouling will fail in clinical specimens. Conversely, a coating that places the analyte outside the plasmonic near field can suppress the optical response it was intended to protect [25,65].

Electron-beam lithography and focused-ion-beam milling remain useful for prototype and master-template fabrication because they provide precise control over aperture size, particle geometry, and spacing [58]. Their limitation is throughput. Disposable clinical cartridges require fabrication methods that can produce many chips with similar resonance positions and similar functionalization behavior. Nanoimprint lithography offers one route, as it can replicate nanoscale patterns over larger areas once a high-quality master is prepared [69].

Nanosphere lithography and colloidal self-assembly offer lower-cost routes. Reviews of plasmonic nanobiosensor fabrication and scalable lithographic processes describe how colloidal masks and related large-area patterning methods can generate periodic metallic nanostructures for optical sensing [25,58]. The trade-off is statistical disorder: particle size distribution, vacancy defects, and imperfect packing broaden resonance features and create spatial heterogeneity. For a diagnostic sensor, these defects are not cosmetic. They can alter baseline intensity, hotspot density, and the apparent target concentration.

Recent fluid-interface-assisted colloidal assembly provides direct experimental evidence for how defects and finite domain size affect the optical linewidth of self-assembled plasmonic arrays. Feller et al. prepared non-close-packed Au nanoparticle Bravais lattices by self-assembly at an air/water interface and transfer to glass substrates. Across six positions on a cm^2^-scale substrate, the average particle density was 5.2 ± 0.3 particles/µm^2^ (less than 10% variation in particle number per unit area) and the plasmon peak position was 584 ± 1 nm. When experimental spectra were compared with defect-free FDTD calculations, differences in peak position and width were attributed to defects and limited domain sizes. After plasma removal of the microgel shell and application of a homogeneous polymer overlayer, the resulting SLR occurred at 617 nm with a FWHM of 40 nm and Q = 15; the authors explicitly attributed the limited Q factor to finite domain size and defects [70]. These data directly support a self-assembled defect/domain-size–linewidth relationship, but they should not be interpreted as evidence of lot-to-lot calibration transfer for a functionalized clinical biosensor.

Surface functionalization usually starts with stable attachment of recognition elements. On plasmonic surfaces, receptor density, spacer composition, and backfilling should be optimized so that target recognition or reporter generation remains accessible to the optical near field [25,61]. Adjacent electrochemical and porous-silicon biosensor studies demonstrate a general trade-off between suppressing nonspecific adsorption and preserving target binding or bioconjugation performance [62,63,64]. For EV assays, capture markers and separation methods also determine which vesicle subpopulation is measured [42,44].

Antifouling chemistry is a central design requirement for non-invasive biofluids. Urine contains salts, urea, creatinine, proteins, cells, and variable pH [14,16]. Saliva contains mucins, proteases, microorganisms, and food- or hygiene-related background [15]. Studies on adjacent electrochemical and porous-silicon biosensors show that PEGylated, zwitterionic, and peptide-based layers can reduce nonspecific adsorption in complex media [62,63,64]. Transfer of these chemistries to plasmonic gold or silver arrays must still be validated in the intended urine or saliva matrix.

The surface layer must be matched to the optical readout. In LSPR, the refractive-index perturbation decays with distance from the metal, so thick surface layers can reduce sensitivity [25,65]. In SERS, the target or reporter must remain close enough to the metal surface for enhancement, whereas MEF requires spacing that permits field coupling without dominant quenching [65,66]. Surface chemistry is therefore an integral part of optical design.

Manufacturing qualification should cover structural, optical, and biochemical variation. Structural quality control can include feature size, array pitch, roughness, and defect density [57,58,69]. Optical quality control should include resonance wavelength, linewidth, and spatial variation across the chip [56,57]. Receptor loading, nonspecific adsorption, blank-matrix response, spiked recovery, and storage stability should be measured on the final plasmonic substrate [62,63,64]. These measurements are needed to determine whether calibration is transferable across chip positions, fabrication lots, and patient samples.

**Figure 3:**
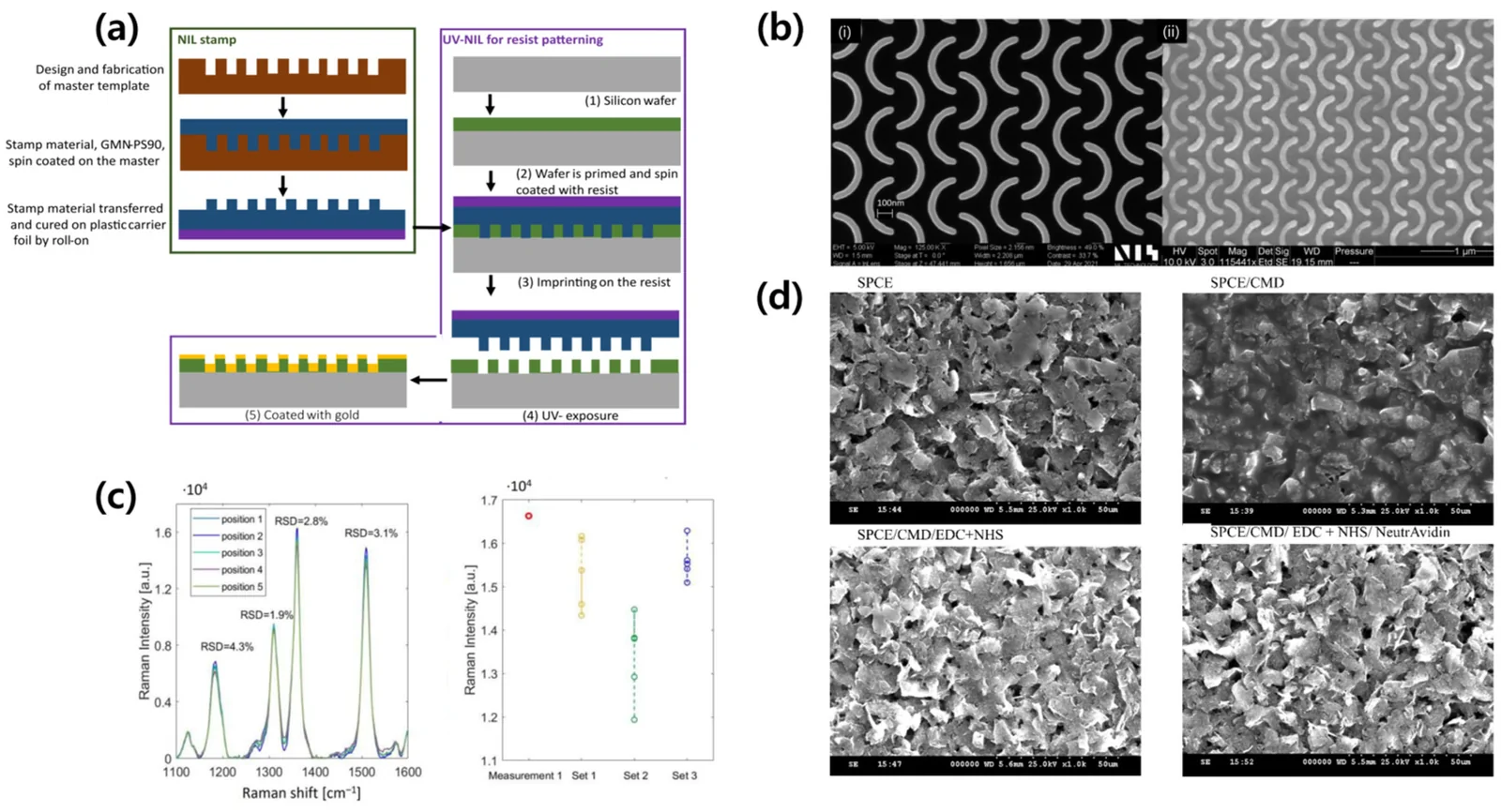
Fabrication and surface-functionalization strategies for biosensor platforms. (**a**) UV-nanoimprint lithography process used to fabricate predefined SERS nanopatterns. Adapted from Ref. [69]. © 2023 by the authors. (**b**) Top-view SEM images of (**i**) the C-shaped silicon master and (**ii**) the corresponding gold-coated UV-NIL replica. Adapted from Ref. [69]. © 2023 by the authors. (**c**) SERS spectra of dried 10 µM Rhodamine 6G measured at five positions on a single chip, showing within-chip signal uniformity (RSD < 4.3% for peaks in the 1100–1600 cm^−1^ range), together with the Raman intensity of the 1361 cm^−1^ peak across repeated detection–cleaning cycles on the same substrate (RSD 8.4%). Adapted from Ref. [69]. © 2023 by the authors. (**d**) For comparison, SEM images of a non-plasmonic SPCE after stepwise modification: bare SPCE, carboxymethyl dextran coating, EDC/NHS activation, and NeutrAvidin immobilization. Adapted from Ref. [34]. © 2022 by the authors.

Reproducibility should also be reported at the scale actually tested. In the UV-NIL SERS substrate already discussed in Figure 3, the RSD < 4.3% corresponds to five measurement positions on a single chip and the 8.4% value corresponds to repeated detection-cleaning cycles on the same substrate [69]; neither is an inter-batch or wafer-scale metric. A 2024 Au nanoTriangle array-on-gel study reported SERS RSD values below 10% across five different batches, including 8.1% at 949 cm^−1^ and 3.2% at 1016 cm^−1^, providing a distinct batch-level benchmark [71]. At larger manufacturing scales, microbubble-assisted replication has produced quasi-3D plasmonic nanostructures across a 4-inch wafer in less than 10 min [72]. AAO-template-assisted evaporation has separately demonstrated centimeter-scale Au and Ag nanoarrays while systematically varying particle diameter (~40–80 nm), deposition thickness (20–50 nm), morphology, and surrounding refractive index (n = 1.0–1.6) [67]. These results represent different levels of reproducibility and manufacturing scale.

Zhou et al. provided a complementary lithographic example using a defect-tolerant cylindrical surface-lattice-resonance (SLR) array. They reported an experimental FWHM of 5.1 ± 1.1 nm, a Q factor up to 154, small resonance-wavelength variations among different batches of successively fabricated arrays, and consistent spectra at three randomly selected locations of a 2 × 2 mm active array; simulations further evaluated breach, bugle, dome, and related structural defects [73]. This study provides defect-tolerance and batch-replication evidence for a lithographic array, whereas Ref. [70] provides the direct self-assembled defect/domain-size-linewidth evidence. Neither result alone establishes calibration transfer for a fully functionalized urine- or saliva-facing diagnostic cartridge.

For nucleic-acid assays, surface density is a major design variable. Capture density, spacer length, backfilling chemistry, and mixed monolayers should be reported because they determine whether short miRNA targets or reporter probes can access the surface without excessive background [25,61]. For protein and EV assays, functional receptor density and capture selectivity should also be reported. EV literature emphasizes that capture markers and separation methods define the measured subpopulation [42,44], while antifouling/bioconjugation studies show that stronger passivation can reduce both specific binding and fouling [64].

Surface-interface reporting should likewise be quantitative whenever the data are available. Grafting density, coating thickness in the hydrated state, spacer length, receptor coverage, and target size together determine the effective target-to-metal distance. Yet, these quantities are not reported consistently across the studies considered here. Because this distance affects LSPR evanescent sensitivity, SERS hotspot accessibility, and MEF enhancement-quenching balance in different ways, missing thickness or density values prevent a rigorous cross-study correction. Where coating thickness or density was not measured directly, these values were treated as not reported rather than estimated from the nominal surface chemistry [62,63,64,74].

A recent plasmonic SERS aggregate study provides a quantitative interface example, although it is not a patterned nanoarray. Potter et al. kinetically arrested AuNP–cucurbit [7]uril aggregates with thiolated PEG; for 40 nm AuNPs, 800 PEG-SH chains per particle corresponded to a grafting density of 0.166 PEG nm^−2^. Aggregate stability and disassembly depended on PEG chain length and grafting density, and 0.166 PEG nm^−2^ was defined in that system as the grafting density sufficient to stabilize aggregates for at least 60 min across the tested PEG molecular weights. The PEG-stabilized aggregates retained SERS detection capability in simulated urine and other salt- or protein-containing media [75]. This adjacent plasmonic evidence supports explicit reporting of ligand chain length and grafting density, but it does not define a universal coating thickness or optimum density for planar urine- or saliva-facing nanoarrays.

Storage is another underreported surface-engineering issue. A disposable cartridge may be fabricated weeks or months before use, and the optical resonance can remain stable even when the biological receptor layer has partially lost activity. Existing fabrication and antifouling studies establish optical uniformity and interfacial performance [57,62,63], but they do not by themselves establish long-term receptor stability in a finished cartridge. Translational studies should therefore measure dry storage, rehydration recovery, and receptor stability alongside optical uniformity. This is especially important for decentralized urine or saliva testing because sample collection and testing may occur outside a specialized laboratory.

Surface validation should use the fabrication route intended for scale-up. Results obtained on an electron-beam-written research chip do not automatically transfer to a nanoimprinted or self-assembled disposable chip. Roughness, grain structure, and defect density can alter optical response [57,58,69], while nonspecific adsorption depends on the final surface chemistry [62,63,64]. Fabrication, functionalization, and biomarker testing should therefore be assessed on the same substrate batch rather than on separately optimized materials.

For a producible disposable cartridge, structural scale-up is only one qualification step. The final functionalized device should additionally report lot-to-lot optical calibration, receptor loading, dry- or wet-storage stability, rehydration recovery, matrix recovery, and failure rates. Where these data are not reported, they should be stated as unavailable rather than inferred from pattern fidelity or optical uniformity. This distinction separates a research-grade chip that demonstrates an optical principle from a manufacturable diagnostic cartridge that must retain both optical calibration and biochemical function during storage, transport, and patient-sample exposure.

Fabrication and surface chemistry should be evaluated together because both affect the measured signal. Pattern fidelity and scalable fabrication determine resonance reproducibility [57,58]. Nanoimprint replication provides a high-throughput route for pattern transfer [69], but calibration transfer across chips must be tested experimentally. Receptor immobilization, hydration, and antifouling chemistry can alter both specific capture and background adsorption [62,63,64], and these effects must be measured on the final urine- or saliva-facing array.

Joint reporting of optical characterization and biofluid performance is needed because the two are not independent.

In practice, the surface design should be finalized only after the assay sequence is fixed. A capture layer that appears stable during static incubation may behave differently under flow conditions. Because urine and saliva differ substantially in composition [14,15], a coating that resists serum proteins cannot be assumed to resist mucins or urinary debris. Matrix-only controls should therefore be run on every surface chemistry considered for translation.

For the same reason, surface-engineering evidence should come from the final urine- or saliva-facing nanoarray. Scaled fabrication and direct exposure to urine or saliva impose conditions not captured by separate buffer-only antifouling or fabrication tests [14,15,69]. A translational report should clearly state which surface was used for the clinical matrix experiment, how it was stored, and whether receptor activity was assessed after fabrication. Otherwise, apparent surface robustness may reflect the test condition rather than the cartridge that will actually contact patient specimens.

Milenko et al. reported the UV-nanoimprint fabrication sequence for predefined SERS nanopatterns (Figure 3a), top-view SEM images of the C-shaped silicon master and the corresponding gold-coated UV-NIL replica (Figure 3b), and within-chip signal uniformity and stability over repeated detection–cleaning cycles demonstrated using Rhodamine 6G (Figure 3c) [69]. As a non-plasmonic comparison, Chiou et al. presented SEM images of the stepwise SPCE modification from the bare electrode through carboxymethyl dextran coating, EDC/NHS activation, and NeutrAvidin immobilization (Figure 3d) [34].

### 3.3. Signal Generation Strategies

LSPR is the most direct label-free readout used in plasmonic nanoarrays. Target binding within the evanescent sensing volume changes local refractive index and shifts resonance wavelength or intensity [25,76]. SPR/LSPR platforms can measure biomolecular interactions without secondary labels, including medical analytes in complex matrices [77,78]. The drawback is that nonspecific adsorption produces the same physical type of signal as specific binding. Urine and saliva testing therefore requires matrix controls, antifouling surfaces, and washing protocols [14,15].

For LSPR, matrix interference can be expressed as competition between the target-induced resonance change and baseline shifts produced by the specimen and interface. Nonspecific protein, mucin, EV, or debris adsorption within the evanescent sensing volume changes the local dielectric environment by the same physical mechanism as specific binding, thereby reducing effective signal-to-background discrimination. Thick passivation or recognition layers can suppress fouling but can also displace the binding event from the highest-sensitivity region. Bulk refractive-index sensitivity can be written as S_RI_ = Δλ_res_/Δn, and a commonly used spectral figure of merit is FOM = S_RI_/FWHM. These quantities describe how strongly and sharply the resonance responds to a refractive-index change, but they are not equivalent to biochemical assay LOD. A higher S_RI_ and narrower linewidth can improve discrimination of small optical shifts; however, assay LOD additionally depends on receptor affinity and density, nonspecific adsorption, instrument resolution, baseline noise, and sample preparation. Recent Au/Ag nanoarray data show that particle diameter, deposition thickness, morphology, and dielectric environment alter resonance position, linewidth, and refractive-index sensitivity, illustrating why optical FOM and biochemical detection performance should be reported separately [67,76].

SERS reads the sample differently. Rather than measuring only refractive index, it amplifies vibrational spectra from molecules or Raman reporters located in plasmonic hotspots [79,80]. That enables multiplexing because different reporters can generate distinct spectral peaks. It can also support label-free biofluid fingerprinting, although those spectra mix disease biology with matrix background [79,80]. A 2026 review similarly notes that urine, saliva, and other biofluids are promising for label-free SERS liquid biopsy but still require careful clinical validation [80].

For SERS, the critical biological-interface variable is access to the electromagnetic hotspot. In the common electromagnetic description, Raman enhancement is related to the local-field gain at the excitation and Raman-shifted frequencies and is often approximated by an |E/E0|^4^ dependence for modest Stokes shifts [81,82]. The field decays steeply away from a hotspot, but a universal r^−12^ distance law should not be assigned to patterned nanoarrays because the decay profile depends on gap geometry, particle shape, coupling mode, and dielectric environment. Thick protein layers, passivation films, or vesicle dimensions can therefore reduce effective SERS gain even when the bare substrate gives a very high simulated hotspot. Protein-delivery experiments in nanogap SERS systems illustrate this accessibility problem directly and show that bringing macromolecular targets into the hotspot can be as important as increasing the nominal field enhancement [71]. EF values should likewise be compared only when their definitions and reference conditions are stated, because maximum, average, and experimentally estimated EFs are not interchangeable [82].

For nucleic-acid targets, SERS can be combined with amplification or sequence-specific recognition. SERS miRNA assays use hybridization, catalytic amplification, and reporter-coded readout [61]. Wang et al. combined a gold nanohexagon array with hybridization chain reaction amplification for salivary oral-cancer-related miRNAs [83]. Linh et al. combined a three-dimensional plasmonic paper substrate with label-free urine spectra and deep-learning-assisted classification [84]. Both studies demonstrate technical and small-cohort feasibility, not readiness for routine clinical testing.

MEF is useful when the assay already relies on fluorescence. Metal nanostructures can increase excitation and radiative decay rates, but enhancement and quenching depend strongly on fluorophore-metal distance [65,66]. Porous gold nanorods have been used for multiplex miRNA fluorescence enhancement [55], and a portable paper-based MEF biosensor was used for CEACAM5 detection [85]. Neither study establishes clinical performance in urine or saliva, where autofluorescence, quenching, and fouling require direct evaluation.

MEF is governed by a related but distinct distance-dependent competition. The observed fluorescence reflects the combined effects of plasmon-enhanced excitation, changes in the fluorophore radiative decay rate, and nonradiative energy transfer or loss to the metal. At very short fluorophore-metal separations, nonradiative pathways can dominate and quench emission; at an appropriate spacer distance and spectral overlap, increased excitation and radiative decay can dominate and enhance fluorescence [74]. Recent mechanistic review data illustrate why this balance must be treated quantitatively but not as a universal spacer rule: quenching in a flat-Au model becomes pronounced below approximately 15 nm, while reported optimum separations differ substantially between specific systems, including about 5 nm for Nile Blue coupled to an 80 nm Au nanoparticle and about 30 nm for DiIC1(5) near a continuous Au film [74]. These values are system-specific examples, not transferable design constants. For urine- or saliva-facing assays, metal geometry, fluorophore spectrum and orientation, quantum yield, recognition-layer thickness, antifouling coating, target size, and the local dielectric environment all contribute to the effective enhancement-quenching balance.

CRISPR-assisted sensing adds sequence specificity before readout. Cas12a nanoparticle biosensors and Cas13a assays for exosomal RNA exploit trans- and collateral cleavage, as well as RNA recognition, to generate sensitive signals [86,87]. Electrochemical amplification cascades and dual-mode luminescence/colorimetric Cas12a implementations demonstrate the range of programmable nucleic acid readouts [88,89]. A CRISPR-Cas12a SERS nanoplatform shows how nuclease recognition can be linked to Raman readout [90]. These examples support trace nucleic-acid detection, but real biofluids can contain inhibitors, nucleases, and ionic conditions that affect enzyme activity [14,15].

Readout choice should follow the clinical question. LSPR is useful for direct binding and has been applied to adjacent EV and serum protein-marker sensing platforms [28,52,78]. SERS supports multiplex reporters and spectral fingerprints. MEF is appropriate when fluorescence assays need signal gain. CRISPR-assisted formats can improve nucleic-acid specificity. In each case, the endpoint is reproducible quantification in urine or saliva, not buffer sensitivity alone [14,15].

The three optical readouts also differ in how easily they support multiplexing. LSPR multiplexing usually relies on spatially separated capture regions or spectrally distinguishable resonances in patterned plasmonic systems [23,91]. SERS multiplexing can use Raman reporters with narrow, non-overlapping bands, which is useful when several miRNAs or protein markers must be measured from a single specimen [61,79]. MEF multiplexing can use spectrally distinct fluorophores, but the enhancement factor depends on fluorophore-metal distance and local optical background [55,65,66]. These differences should guide assay design before the biomarker panel is finalized.

Negative samples create different failure modes for each readout. In LSPR, a valid negative control should remain stable after matrix exposure and washing because nonspecific refractive-index changes generate the same physical readout as target binding [25]. In SERS, endogenous biofluid spectra can remain information-rich even in the absence of the target, so classification models must separate disease patterns from matrix effects [79,80]. In MEF, autofluorescence or quenching can affect signal even without target binding [65,66].

For CRISPR-assisted formats, the most important validation is not only whether the Cas reaction works in buffer. Urine and saliva can contain nucleases, changes in divalent ion levels, pH variations, and inhibitors that alter guide RNA stability or collateral cleavage activity [14,15]. Cas12a nanoparticle biosensing and Cas13a exosomal-miRNA assays illustrate the trans/collateral-cleavage and RNA-recognition principles used in these applications [86,87]. Electrochemical amplification cascades, dual-mode luminescence/colorimetric systems, and SERS-coupled platforms extend them to distinct readout modalities [88,89,90].

The readout modes differ in signal origin, multiplexing strategy, and matrix sensitivity. LSPR offers label-free detection but is sensitive to nonspecific refractive-index shifts [25]. SERS enables multiplex spectral readout, although access to hotspots and statistical validation are important [80]. MEF can increase fluorescence signals but depends strongly on fluorophore-metal distance [65,66]. CRISPR-assisted formats add sequence specificity, while matrix effects on enzyme activity still have to be tested [86,87,90].

For urine- and saliva-based diagnostics, performance should ultimately be evaluated in the intended matrix. The completed nanoarray should preserve target-specific signal after functionalization, sample exposure, washing, and repeated readout in the intended urine or saliva specimen. SEM images, narrow resonances, and hotspot simulations do not substitute for recovery, background, and repeatability data in authentic biofluids. Reports should state whether measurements were performed in buffer, artificial matrix, diluted clinical specimens, or untreated clinical urine or saliva because these conditions impose different transport, fouling, and background burdens [14,15].

**Figure 4:**
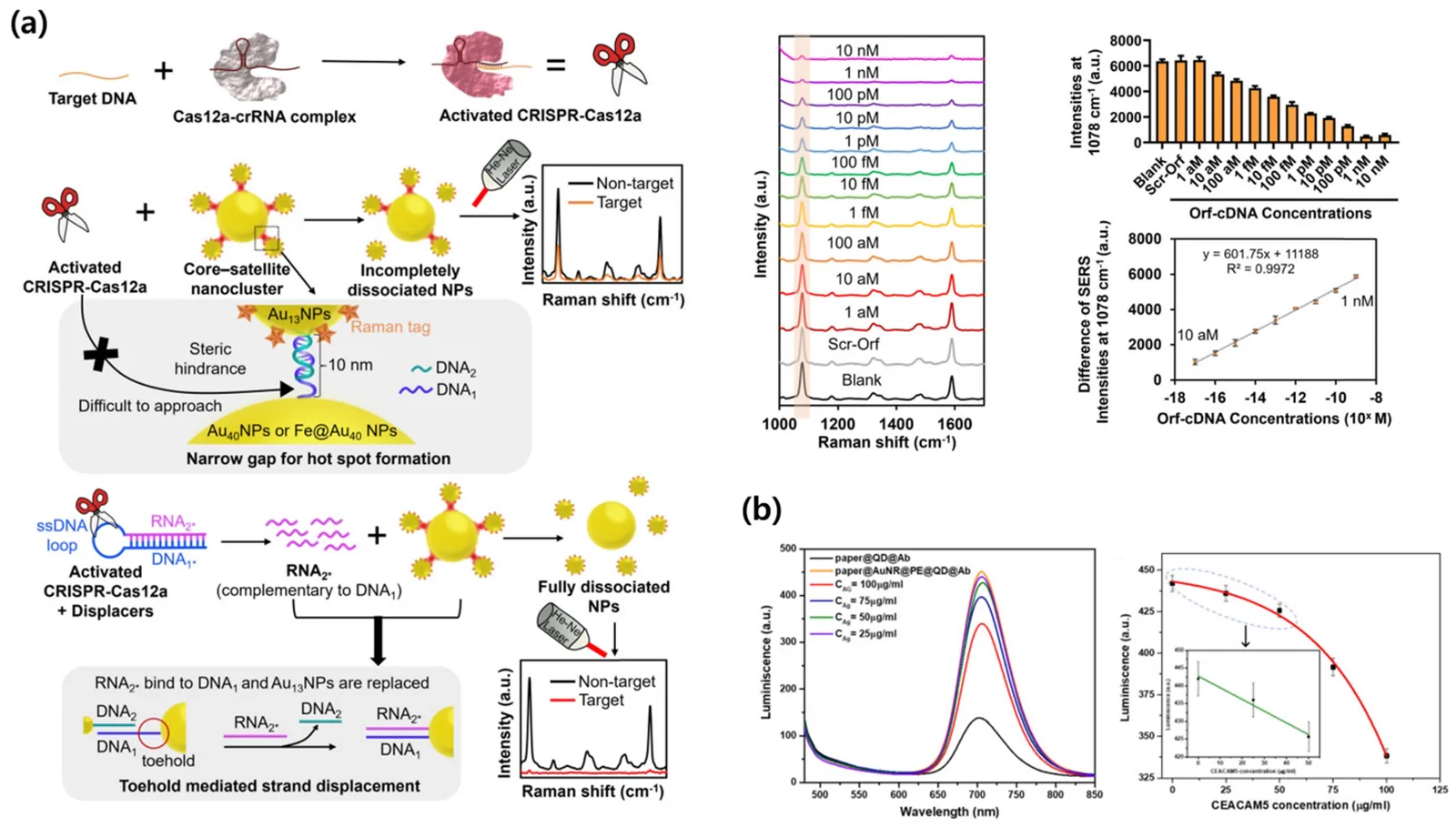
Plasmonic signal-generation strategies. (**a**) CRISPR-Cas12a/displacer-mediated dissociation of core–satellite nanoclusters and the corresponding concentration-dependent SERS response to SARS-CoV-2 Orf-cDNA, used here as a proof-of-concept nucleic-acid target for CRISPR-coupled plasmonic signal generation. Adapted from Ref. [90]. © The author(s). (**b**) Luminescence spectra and decreasing luminescence and MEF responses with increasing CEACAM5 concentration during indirect detection using antibody-conjugated quantum dots on a paper-based AuNR platform. Adapted from Ref. [85]. © 2022 by the authors.

CRISPR-mediated target recognition can be converted into a SERS response through changes in nanoparticle assembly. Yin et al. designed a Cas12a-based assay in which target-activated Cas12a cleaved the loop of a chimeric DNA/RNA displacer. The released RNA strand then dissociated the core–satellite nanoclusters through toehold-mediated strand displacement, reducing the SERS signal at 1078 cm^−1^ as the Orf-cDNA concentration increased (Figure 4a) [90]. The viral sequence was used as a proof-of-concept target, and the platform is discussed here as an example of CRISPR-coupled plasmonic signal generation. Susu et al. used antibody-conjugated quantum dots on a paper-based AuNR platform for indirect CEACAM5 detection. The luminescence intensity decreased with increasing CEACAM5 concentration (Figure 4b) [85].

## 4. Clinical Applications

### 4.1. Platform Integration for Clinical Translation

In clinical use, a plasmonic nanoarray test would need to operate as a sample-to-answer workflow rather than only as a sensing surface. Microfluidics can control sample volume, flow rate, incubation time, reagent sequence, and washing conditions [92,93,94]. Microfluidic plasmonic and SERS literature treats transport, mixing, surface exposure, washing, and optical readout as coupled design problems rather than independent modules [91,95].

Preprocessing is matrix-specific. Urine may require removal of cells and debris, dilution control, ionic strength adjustment or EV enrichment before the sample reaches the sensing region [14,16]. Saliva may require viscosity control, debris removal, controlled dilution, and standardized collection or processing to improve transport and reproducibility [15]. EV assays need particular care because the measured population depends on the isolation or capture method [42,44]. A 2026 digital droplet microfluidic platform integrated compartmentalization with simultaneous profiling of single-exosome surface proteins and miRNAs [96]. Microfluidic SERS reviews make a related point regarding cancer biomarkers: sample handling and optical readout must be designed together [95].

Portable optical readout is another requirement for translation. Mobile nucleic acid diagnostic reviews and portable metasurface-plasmon platforms indicate that analytical workflows and optical measurements can shift toward compact readers [68,97]. Reviews of compact microfluidic biosensing platforms and multiplexed point-of-care testing also show how multi-analyte measurement can move toward decentralized formats [98,99]. Multiplexing becomes useful when the panel is clinically coherent and tied to a prespecified decision; adding unrelated channels does not make a diagnostic test [99]. In cancer applications, the final output should be a diagnostic or risk score used for biopsy triage, recurrence surveillance, or imaging referral.

Quality-control regions should be incorporated into the cartridge. A matrix-background region can reveal nonspecific adsorption, a positive-control region can confirm reagent activity, and a flow-control region can identify inadequate sample delivery or washing. These controls are particularly important for self-collected specimens with low volume, high viscosity, food contamination, hematuria, or delayed processing [14,15]. Reporting guidelines for diagnostic-accuracy studies and clinical AI also require prespecified endpoints, transparent error analysis, and external validation when computational interpretation is used [100,101,102].

The integration strategy should also define how much processing is acceptable in the intended setting. A laboratory assay may tolerate centrifugation, filtration, and multiple incubation steps, but a point-of-care urine or saliva test should minimize manual operations [92,94,97]. If EV enrichment is required, the enrichment module must preserve the vesicle population relevant to the diagnostic claim [42,44]. If nucleic acid amplification or CRISPR recognition is included, buffer composition and reaction time must be controlled; matrix compatibility should be tested directly, as the cited CRISPR platforms are primarily technical, buffer-based, or plasma-based demonstrations rather than validated urine/saliva workflows [86,87,88,89,90].

Clinical translation also requires a clear comparator. For bladder cancer surveillance, clinically relevant reference procedures include cystoscopy and cytology; urine studies for tumor DNA, methylation, miRNA, cfDNA, and SERS provide candidate adjunctive molecular evidence rather than replacement validation [9,48,103,104,105]. For prostate cancer, clinically relevant comparators include biopsy outcome and clinically significant disease; current urinary EV-miRNA studies provide screening and progression-assessment evidence rather than validated biopsy-triage performance [33,34,35]. AI-assisted urinary EV approaches provide additional diagnostic and classification evidence [106]. For oral cancer, healthy controls alone are insufficient because benign inflammatory, periodontal, and infectious conditions can alter saliva and mimic disease-associated background [15,19].

Failure handling should be predefined. Some point-of-care specimens will be too viscous, too dilute, blood-contaminated, or delayed in processing. Such samples should be reported as invalid or high-background rather than forced into a positive or negative category [14,15]. When a classifier or prediction model interprets the readout, the failure rules and deployment limits should also be prespecified [100,101,102].

A peak shift or integrated Raman intensity alone cannot distinguish target binding from bulk matrix drift or incomplete washing [25,80]. A clinically oriented reader should report the biomarker signal together with quality-control flags such as high background, a weak positive control, or failed flow. AI-specific reporting guidance applies when a classifier or prediction model converts these measurements into a clinical result [100,101,102].

**Figure 5:**
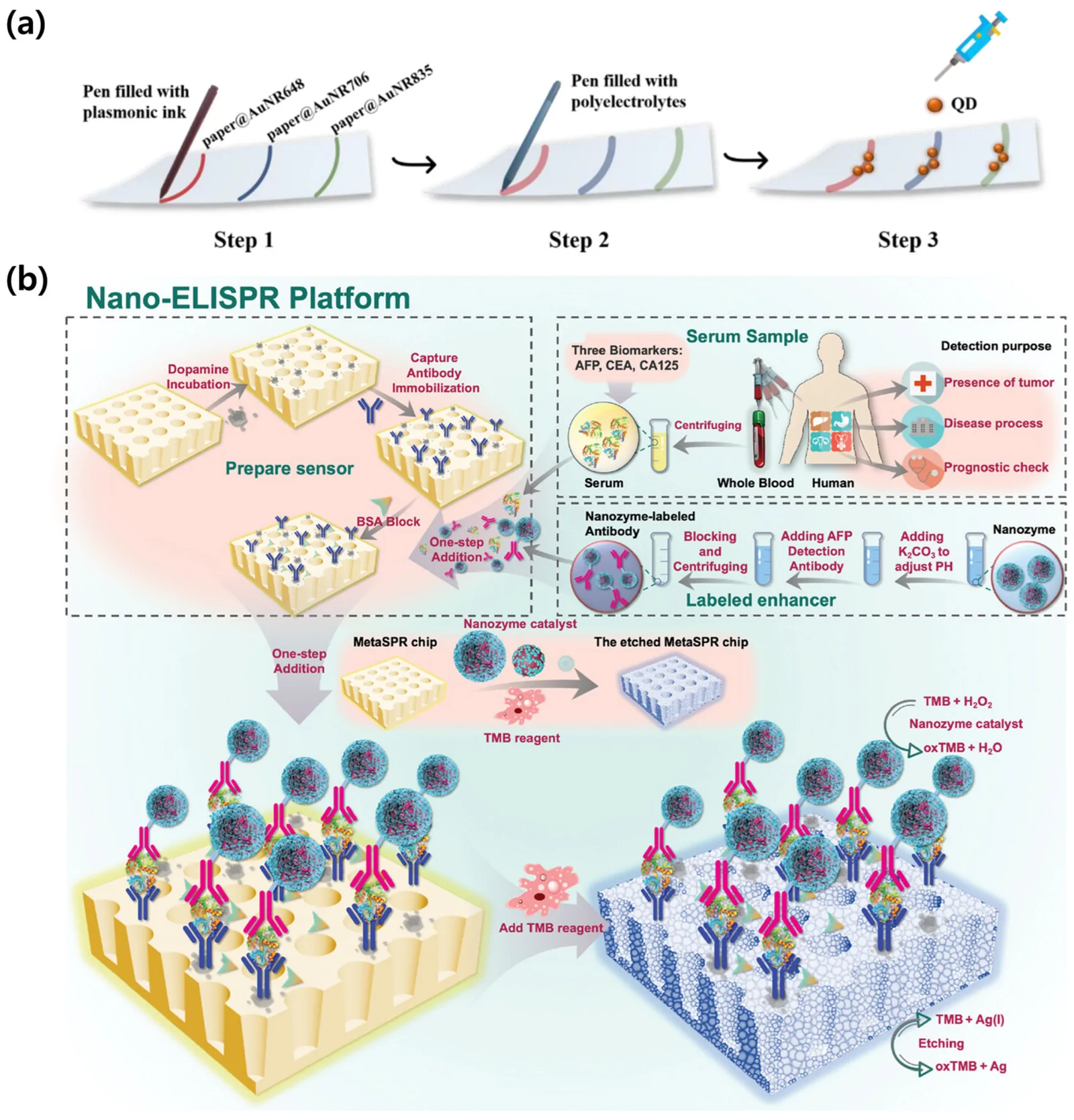
Portable plasmonic assay formats. (**a**) Fabrication of spatially isolated AuNR lines on filter paper by plasmonic calligraphy, followed by PSS/PAH coating and quantum-dot deposition. Adapted from Ref. [85]. © 2022 by the authors. (**b**) Nano-ELISPR workflow using a gold–silver nanocup metasurface and nanozyme-labeled antibodies for the detection of AFP, CEA, and CA125 in serum. Adapted from Ref. [68]. © 2023 The Authors.

Susu et al. prepared spatially isolated AuNR lines on filter paper by plasmonic calligraphy, coated them with PSS and PAH spacer layers, and deposited quantum dots on the resulting substrates. The paper-based MEF platform was subsequently used for indirect CEACAM5 detection (Figure 5a) [85]. Li et al. developed a Nano-ELISPR platform that combined a gold–silver nanocup metasurface with nanozyme-labeled antibodies to quantify AFP, CEA, and CA125 in serum (Figure 5b) [68]. Neither platform was evaluated in urine or saliva, but both show how plasmonic substrates, assay chemistry, and optical readout can be combined in portable sensing formats.

### 4.2. Cancer-Type-Specific Applications

The clinical value of a urine- or saliva-based plasmonic nanoarray platform depends on cancer type. The rationale is strongest when the biofluid is anatomically or biologically connected to the disease site, and the assay answers a defined clinical question. Bladder surveillance, prostate biopsy triage, oral cancer referral, and high-risk pancreatic monitoring require different biomarker panels, comparators, and validation strategies.

**Figure 6:**
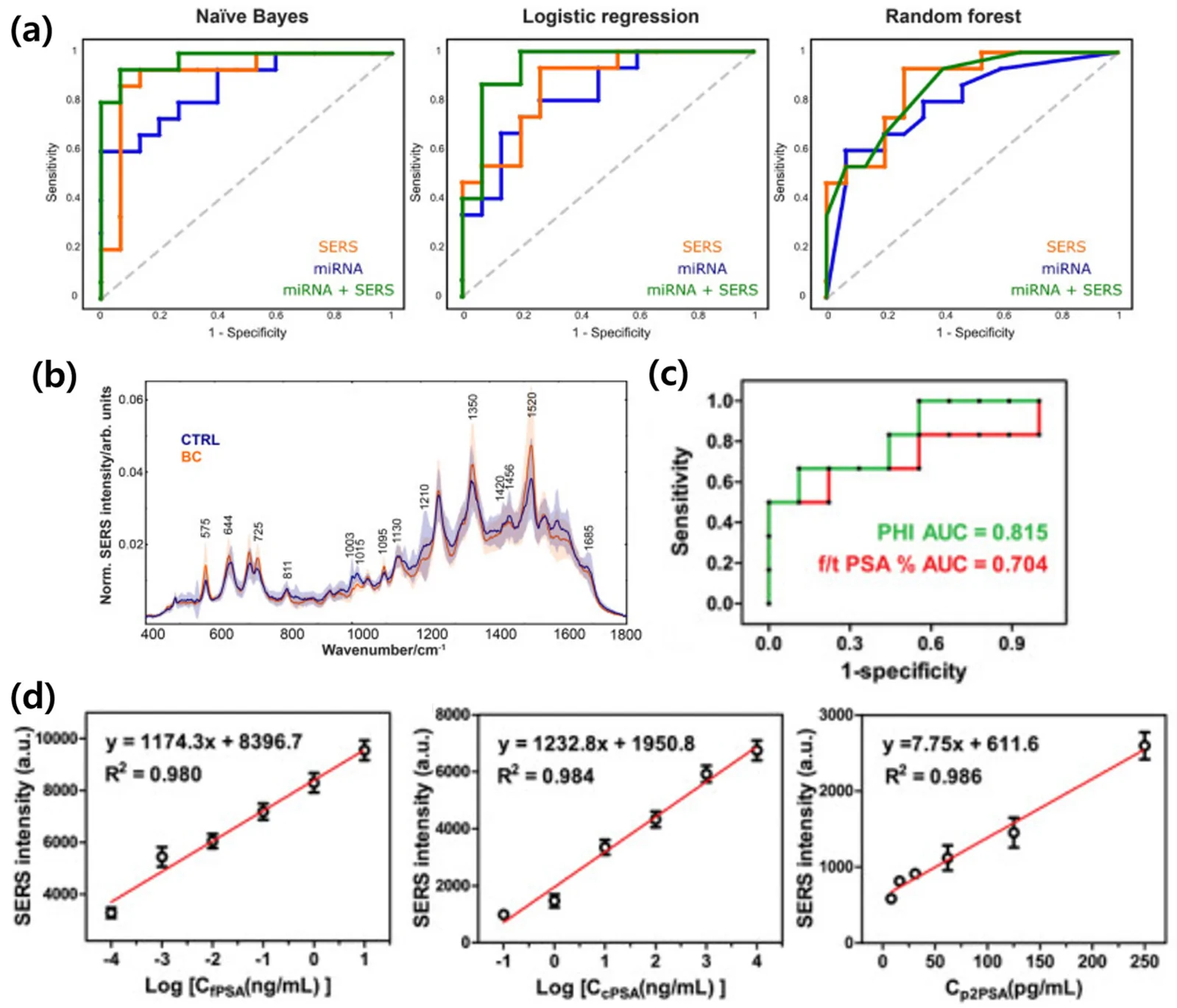
Urine- and serum-based plasmonic assays for bladder and prostate cancer assessment. (**a**) ROC curves obtained using urinary SERS data, miRNA expression, and combined miRNA–SERS models with naïve Bayes, logistic regression, and random forest classifiers. Adapted from Ref. [48]. © The Author(s) 2022. (**b**) Mean urinary SERS spectra of patients with bladder cancer and control participants. Adapted from Ref. [48]. © The Author(s) 2022. (**c**) ROC comparison of the prostate health index (PHI; AUC = 0.815) and the percentage of free-to-total PSA (f/t PSA%; AUC = 0.704) in clinical serum samples. Adapted from Ref. [45]. © 2024 The Author(s). (**d**) Calibration curves for the SERS detection of free PSA (fPSA), complexed PSA (cPSA), and [−2]proPSA (p2PSA) in 1% serum. Adapted from Ref. [45]. © 2024 The Author(s).

Bladder cancer is directly linked to urine because urothelial tumors can release tumor-derived molecular material into the urinary tract. Urinary tumor DNA has been investigated for detection and surveillance [9], and studies of urinary miRNAs, cfDNA, and DNA methylation support molecular urine testing [103,104,105]. Moisoiu et al. combined urinary miRNA measurements with SERS profiles and reported ROC-based bladder cancer classification using miRNA, SERS, and combined models (Figure 6a) [48]. These data support urine-based molecular testing but do not establish replacement of cystoscopy or cytology.

For bladder cancer, cfDNA methylation is particularly relevant because methylation markers can provide cancer-associated molecular specificity in a urine matrix [103]. Plasmonic nanoarrays could contribute by improving signal amplification, miniaturization, and multiplexed capture of methylated DNA or miRNA targets; EV-associated targets would require separate bladder-specific validation. However, many bladder studies are still molecular-biology or spectroscopy demonstrations rather than fully integrated nanoarray cartridges. The key translational question is whether a chip can detect clinically meaningful recurrence or high-risk disease while controlling for hematuria, infection, and inflammatory background [14,16]. Table 2 therefore separates analytical feasibility from clinical validation status.

Prostate-cancer assays require clinically relevant endpoints beyond the presence of cancer. The intended use may be detection of clinically significant disease, biopsy triage, or progression assessment. Long-term follow-up of randomized PSA-screening trials shows a modest mortality benefit alongside false-positive testing, overdiagnosis, and downstream diagnostic burden [107,108]. Urinary EV-miRNA studies, including a biosensor platform, have evaluated screening, diagnosis, and progression [33,34,35], while AI-assisted urinary EV approaches provide classification evidence without established biopsy-triage utility [106]. Reviews of exosomal miRNAs and blood- and urine-based biomarkers provide additional context, but only part of this literature is linked to established clinical risk-stratification pathways [109,110,111].

Urinary exosomal miRNAs are attractive targets because EV encapsulation can stabilize miRNA cargo and urinary EVs can carry prostate-associated molecular signals [33,35,42]. For the three-dimensional hierarchical SERS platform, analytical calibration in spiked human serum gave an LOD of approximately 10 aM for both miR-10a and miR-21 and a reported linear range of 10 aM–100 nM [112]. Clinical validation was then performed using urinary exosomal miRNAs from 10 patients with prostate cancer and 5 healthy controls (n = 15). The reported ROC AUCs were 0.96 (95% CI, 0.87–1.00) for miR-10a and 0.98 (95% CI, 0.91–1.00) for miR-21, with a diagnostic accuracy of 0.93 for each target [112]. These values describe different stages of evaluation and should not be combined into a single performance metric. Because the cohort was small, the results support feasibility rather than clinical readiness. Larger studies should compare the assay with biopsy outcome, clinically significant disease, PSA density, MRI findings, and established urine molecular tests.

The EV-RNA biochip study profiled EV RNA markers in serum and urine from patients with prostate cancer, benign prostatic hyperplasia, and healthy controls [113]. It supports the biological rationale for urinary EV-RNA panels but is not a plasmonic SERS assay. A separate SERS PHI assay compared PHI with f/t PSA% in clinical serum samples (Figure 6c) and established calibration curves for fPSA, cPSA, and p2PSA in 1% serum (Figure 6d) [45]. This serum study is included as an example of multiplexed plasmonic protein readout and PHI-based risk assessment, not as direct evidence for urine- or saliva-based diagnosis.

Pancreatic cancer has a high unmet need for earlier detection, but its anatomical link to urine or saliva is weaker than that of bladder, prostate, or oral cancer. Serum microRNA signatures have been evaluated as liquid biopsy tools for earlier diagnosis of pancreatic cancer [114]. Recent multicenter evidence shows that urinary extracellular-vesicle miRNA profiles can distinguish pancreatic ductal adenocarcinoma from high-risk conditions and can detect a proportion of early-stage cases [115]. A multicenter blood-based exosome transcriptomic signature provides additional support for EV biomarkers in early pancreatic cancer, but it is neither urine/saliva evidence nor plasmonic nanoarray validation [116]. For pancreatic cancer, the current evidence is better framed around targeted testing in high-risk groups or adjunctive risk assessment than around population screening.

Saliva is particularly relevant to oral and head-and-neck cancers because it contacts oral and oropharyngeal lesions and contains miRNAs, transcripts, proteins, EVs, and microbial or inflammatory signals [10,19,36]. Candidate salivary miRNAs have been evaluated for oral cancer [10,117,118]. Wang et al. reported saliva-matrix LODs of 6.51 aM for miR-31 and 6.52 aM for miR-21 using a gold nanohexagon HCR-SERS array and tested saliva from OSCC patients and healthy participants [83].

For head-and-neck cancer more broadly, label-free SERS biomarker studies and SERS lateral-flow assays for circulating ctDNA provide adjacent optical evidence [119,120]. They are not equivalent to clinical validation in saliva. Inflammation, infection, periodontal disease, smoking, oral hygiene, and recent food intake can all alter the measured salivary background [15,19]. Validation cohorts should therefore include benign inflammatory and infectious controls rather than healthy participants alone. Adjunctive referral or post-treatment surveillance may be practical initial uses because the clinical question is defined and repeated sampling is useful.

Disease-biofluid matching remains central to interpretation. Bladder cancer has the most direct evidence from urinary tumor DNA, DNA methylation, miRNA, and cfDNA [9,48,103,104,105]. Prostate cancer is supported by urinary RNA and EV evidence, although clinically significant disease and biopsy triage remain validation targets rather than established claims [33,34,35,106,109,110,111,112,113]. Oral and head-and-neck cancers are supported by salivary miRNAs and local lesion-associated markers [10,19,36,83]. Pancreatic-cancer evidence should be separated into direct urinary molecular studies and adjacent serum or blood-derived studies [114,115,116]. Any clinical claim must remain tied to the tested biofluid, biomarker, comparator, and patient cohort.

**Figure 7:**
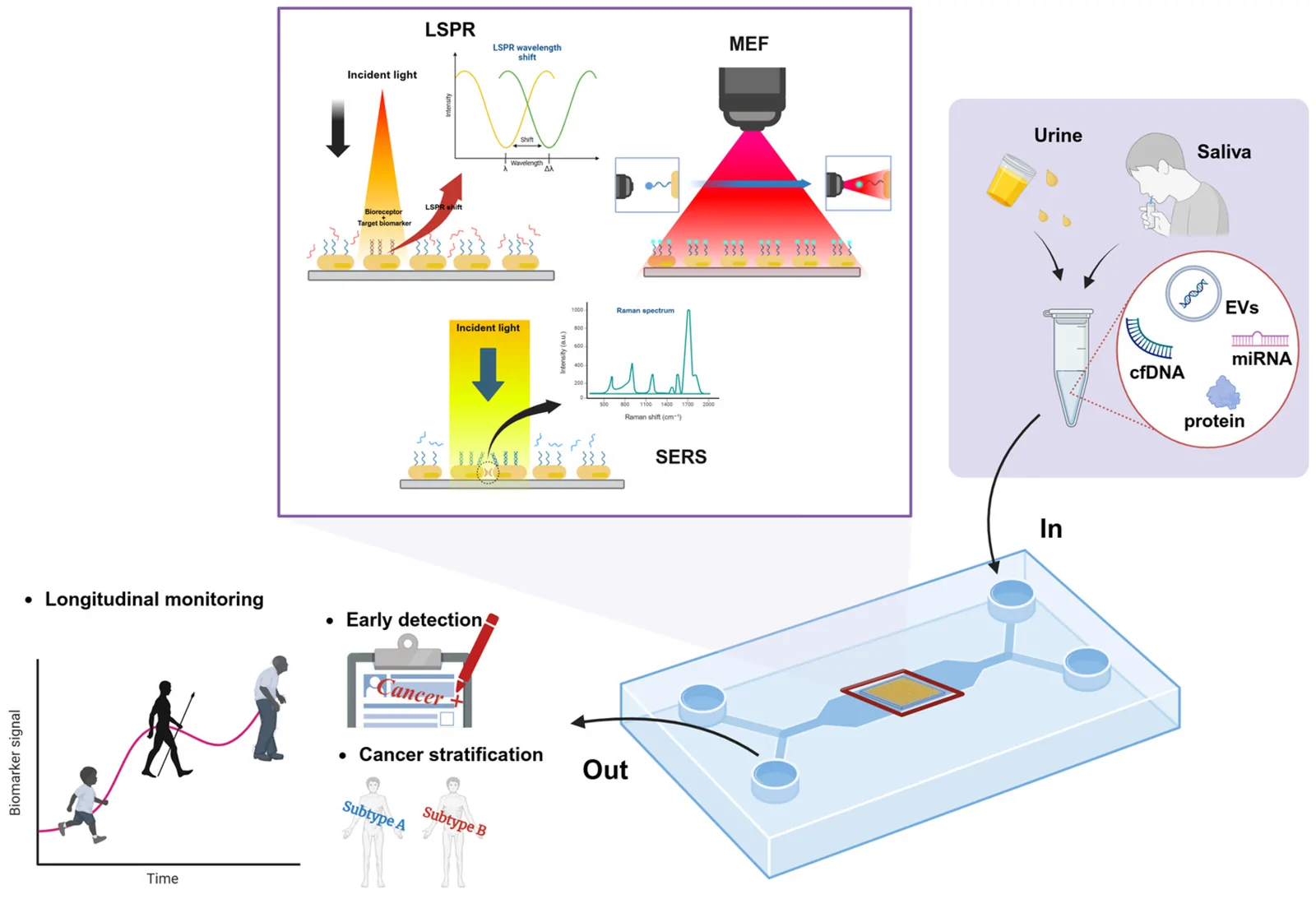
Overall workflow of plasmonic nanoarray-based cancer diagnostics. Urine and saliva provide cancer-associated biomarkers, including EVs, miRNAs, cfDNA, and proteins. Samples are introduced into a microfluidic platform containing a plasmonic nanoarray sensing region. Biomarker recognition is transduced through LSPR, SERS, or MEF optical readouts, supporting early detection, cancer stratification, and longitudinal monitoring.

Moisoiu et al. evaluated bladder cancer classification using urinary miRNA data, SERS profiles, and combined miRNA–SERS models (Figure 6a) and presented the corresponding mean urinary SERS spectra of patients with bladder cancer and control participants (Figure 6b) [48]. For prostate cancer, a multiplex SERS assay compared the diagnostic performance of PHI and f/t PSA% in clinical serum samples (Figure 6c) and provided calibration curves for fPSA, cPSA, and p2PSA in 1% serum (Figure 6d) [45]. The overall matrix-informed diagnostic workflow is summarized in Figure 7.

**Table 2:** Representative cancer-specific studies and supporting non-invasive liquid-biopsy evidence. Analytical performance is reported together with the tested matrix, clinical cohort, diagnostic performance, and validation status.

Cancer Type	Biofluid	Biomarker	Sensing Mechanism/Platform	Analytical Performance	Clinical Cohort/Diagnostic Performance	Validation Status/Limitation
Bladder cancer	Urine	miRNA + urinary molecular/SERS profiles	Combined urine miRNA profiling + label-free SERS classification [48]; adjacent tumor-DNA/cfDNA-methylation evidence [9,103,104,105]	Clinical classification rather than a concentration LOD was the primary endpoint in [48].	Prospective cohort: 15 BC + 16 controls; retrospective miRNA-validation cohort: 66 BC + 50 controls. Combined miRNA + SERS AUC 0.92 ± 0.06 for BC vs controls; luminal/basal AUC 0.95 ± 0.03 [48].	Clinical urine feasibility. The retrospective cohort validated miRNAs, not SERS; not established as a replacement for cystoscopy/cytology.
Prostate cancer	Urine/urinary EVs	Urinary exosomal miR-10a and miR-21	3D hierarchical label-free SERS nano-architecture [112]; supporting urinary EV-miRNA evidence [33,34,35]	Analytical calibration in spiked human serum: ~10 aM LOD for both targets; reported linear range 10 aM–100 nM [112].	Clinical urine: 10 PC patients + 5 healthy controls (n = 15). ROC AUC 0.96 (95% CI, 0.87–1.00) for miR-10a and 0.98 (95% CI, 0.91–1.00) for miR-21; diagnostic accuracy 0.93 for both targets [112].	Small-cohort clinical feasibility; larger biopsy-referenced validation for clinically significant disease is still needed.
Prostate cancer	Serum comparator	fPSA, cPSA, p2PSA for PHI	Dual-enhanced SERS satellite immuno-nanocomplex [45]	1% serum calibration: LOD 0.11 pg/mL fPSA; 0.12 ng/mL cPSA; 0.004 ng/mL p2PSA. Dynamic ranges: 10^−4^–10^1^ ng/mL (fPSA), 10^−1^–10^4^ ng/mL (cPSA), and 0.008–0.25 ng/mL (p2PSA) [45].	Clinical analyses included PCa-versus-healthy identification (N = 13), gray-zone screening (N = 10), and pre/post-surgery monitoring of PCa patients (N = 5). PHI AUC 0.815; f/t PSA% AUC 0.704 [45].	Useful multiplexed plasmonic comparator, but not direct urine/saliva evidence.
Pancreatic cancer	Urine	Urinary EV-miRNA signature	16-miRNA machine-learning urinary EV algorithm [115] (non-plasmonic supporting evidence)	No plasmonic nanoarray LOD; molecular-classification endpoint.	248 PDAC/high-risk samples across multiple Japanese institutions; AUC 0.89, sensitivity 0.80, specificity 0.79; early-stage sensitivity 0.73 [115].	Direct non-invasive urine clinical mapping evidence, but not validation of a plasmonic nanoarray.
Oral/head-and-neck cancer	Saliva	miR-31 and miR-21	Gold nanohexagon array + HCR-SERS [83]; adjacent salivary biomarker evidence [10,117,118]	6.51 aM miR-31; 6.52 aM miR-21 in human saliva [83].	Clinical saliva: 30 OSCC patients + 30 healthy subjects (n = 60). SERS measurements of miR-31 and miR-21 were reported to agree with qRT-PCR [83].	Needs benign inflammatory/infectious controls, multicenter saliva protocols, and external validation.

## 5. Challenges and Future Perspectives

### 5.1. Current Limitations

The principal limitation is the gap between analytical sensitivity and clinical diagnostic performance. The available studies range from buffer-based or technical demonstrations [55,69,85,86,87,88,89,90] and artificial-saliva testing [54] to clinical urine or saliva studies [48,83,84,112]. These conditions are not interchangeable. Translational studies should report recovery in authentic matrices, specificity against realistic benign controls, invalid-test rates, and agreement with a prespecified clinical endpoint. Prediction models should follow appropriate reporting standards [100,101,102], and the intended clinical use should be defined before cohort selection.

As detailed in Section 2.3 and Section 3.2, matrix composition and biofouling are coupled determinants of analyte transport, target accessibility, and optical background rather than independent limitations. Antifouling, recognition efficiency, and optical readout should therefore be evaluated on the same final urine- or saliva-facing interface. Matrix-only controls, target-spike recovery, and post-wash baseline drift should be reported together, and a coating optimized in serum or buffer should not be assumed to retain the same behavior in urine or saliva [14,15,16,18,19,62,63,64,121].

Manufacturing reproducibility is another limitation. Nanoimprint, lithographic, and self-assembly methods can produce large-area arrays, but variation in feature geometry, defect density, and pattern replication can shift resonance position and hotspot density, thereby threatening calibration transfer [57,58,69]. The available quantitative evidence spans different levels and must be labeled accordingly: the RSD < 4.3% in Ref. [69] is within-chip, whereas a 2024 Au nanoTriangle SERS platform reported <10% RSD across five different batches [71]. Microbubble-assisted replication has demonstrated quasi-3D plasmonic structures at 4-inch wafer scale in less than 10 min [72], while AAO-templated evaporation has shown centimeter-scale Au/Ag nanoarray fabrication with geometry-dependent LSPR behavior [67]. These manufacturing demonstrations do not by themselves establish wafer-wide resonance RSD, calibration transfer after receptor immobilization, or clinical-matrix performance. Reports should therefore distinguish within-chip, inter-chip/inter-batch, centimeter-scale, and wafer-scale evidence and should state which level was actually tested. Representative engineering evidence across these reproducibility levels is summarized in Table 3.

Clinical validation remains limited. Many studies use small patient cohorts, healthy controls rather than disease-mimicking controls, or artificial specimens. This is especially problematic for saliva, where inflammation and infection can mimic cancer-associated background, and for urine, where hematuria or urinary tract inflammation can change DNA and protein content [15,16]. Transparent patient selection, prespecified thresholds, independent validation, error analysis, invalid-sample handling, and quality-control reporting are therefore required for translational biosensor studies. When a computational classifier or prediction model is used, TRIPOD+AI, FUTURE-AI, and STARD-AI provide additional model-specific reporting and deployment guidance [100,101,102].

### 5.2. Future Directions

The biofluid and clinical question should be specified before the nanoarray format is selected. Bladder-surveillance assays can build on urine evidence for tumor DNA, DNA methylation, miRNA, and cfDNA and should be compared with cystoscopy-centered follow-up [9,48,103,104,105]. Prostate assays should target clinically significant disease or biopsy triage and can draw on urinary RNA and EV evidence [33,34,35]; AI-assisted EV models currently provide classification benchmarks rather than validated triage tools [106]. Saliva platforms are best aligned with oral and head-and-neck cancer when inflammatory controls are included [10,15]. For pancreatic cancer, direct urinary EV-miRNA evidence in high-risk patients [115] should remain separate from serum or blood-derived evidence [114,116].

Multiplexing is useful only when each channel contributes to the same clinical decision. A panel may combine miRNA, cfDNA methylation, EV capture, and protein readout, but unrelated targets should not be added solely to increase channel count. SERS supports multiplex spectral separation [83], LSPR supports direct binding and EV-oriented plasmonic capture or profiling [25,28,52], and MEF can amplify fluorescence assays [55]. CRISPR-assisted readout can add sequence specificity after matrix compatibility has been established [86,87,90].

CRISPR-assisted optical and nanoplasmonic workflows are being explored for nucleic-acid targets because they can combine programmable recognition with optical signal generation [89,90]. Urine and saliva studies show substantial variation in pH, ionic composition, enzymes, and other matrix components [14,15], but the cited CRISPR platforms do not establish performance in untreated clinical urine or saliva [86,87,89,90]. Future studies therefore need direct matrix-compatibility testing, guide-RNA stability assessment, false-positive cleavage analysis, and reaction-time optimization in authentic specimens.

Computational analysis may help separate weak disease signals from complex SERS or imaging backgrounds [80,84], but it cannot serve as an unreported correction step. Models require external validation, calibration, subgroup analysis, failure-case reporting, and defined deployment limits [101,102,122]. Translation also requires biofluid-specific surface chemistry validated on the final plasmonic array [62,63,64], together with integrated microfluidic handling, multiplexed readout, and quality-control regions [92,95,98,99]. Compact readers provide a route toward decentralized testing [68,98].

Future studies should explicitly report negative and invalid results. Decentralized urine or saliva samples may be too diluted, blood-contaminated, viscous, degraded, or delayed. Treating these as ordinary negatives can inflate accuracy and hide failure modes. Studies should define rejection criteria, background thresholds, control-region behavior, and repeat-test rules before patient testing.

## 6. Conclusions

Plasmonic nanoarrays can combine optical-field enhancement, surface recognition, and small sample volumes, but their diagnostic value depends on the selected biofluid and clinical question [22,25]. Urine-based applications are best supported for bladder and prostate cancer, where tumor-derived or prostate-associated material can enter urine [9,33,34]. Urinary EV-miRNA studies provide detection and progression evidence [33,34], while a serum-and-urine EV-RNA biochip study provides diagnostic profiling but not validated progression or biopsy-triage performance [113]. Saliva is most directly relevant to oral and head-and-neck cancer [10,36]. For pancreatic cancer, emerging urinary evidence [115] must remain distinct from adjacent serum and blood-derived studies [114,116].

Key components have been demonstrated separately, including nanohole sensing and near-aperture trapping [49,50], EV-oriented nanoplasmonic arrays and nanowells [28,52], plasmon-enhanced single-EV fluorescence [53], and scalable lithographic or nanoimprint fabrication [57,58,69]. Antifouling and bioconjugation strategies reported for related biosensor platforms should be validated directly on the final urine- or saliva-facing array [62,63,64]. Additional evidence includes EV and miRNA biology [37,38,42], EV reporting standards [44], single-exosome microfluidic analysis [96], SERS and MEF readout [55,83], microfluidic sample handling [92,93,94,95], and CRISPR-assisted sequence recognition linked to optical readout [86,87,88,89,90].

Clinical translation requires these components to be validated in one sample-to-answer workflow. The same device must handle the matrix, present the target to the nanoarray, suppress nonspecific background, generate an optical signal, and produce an interpretable result. Surface chemistry, preprocessing, optical readout, and the analysis rule should therefore be tested together rather than inferred from separately optimized experiments.

Optical sensitivity and multiplexing are useful only when they are preserved in authentic urine or saliva. Studies should report matrix-matched calibration, recovery, baseline drift, chip-to-chip variation, assay failures, and patient-cohort performance [14,15]. Computational prediction additionally requires transparent model reporting and external validation [100,101,102]. In early-stage disease, where cancer-associated signals may approach the biofluid background, negative controls and matrix-specific validation are as important as the analytical detection limit.

Clinical readiness will depend on prespecified thresholds, realistic benign controls, and independent patient cohorts. Urine and saliva are easy to collect but variable in composition [14,15]; studies should therefore report invalid samples, high-background specimens, and benign conditions that can mimic cancer-associated molecular or optical signals. When a prediction model is used, these elements should be defined and reported according to appropriate diagnostic-AI guidance [100,101,102].

Sensor design should reflect the intended specimen. A urine assay should address dilution, hematuria, salts, urinary proteins, and storage effects [14,16]. A saliva assay should address mucins, proteases, oral microbes, food or hygiene background, and collection variability [15]; disease-associated protease profiles further show that enzymatic composition can vary across clinical conditions [18]. These matrix constraints should determine the capture chemistry, washing step, amplification module, and optical readout.

Nanoarray-based optical assays should be developed as disease- and specimen-specific tools. They must operate under realistic urine or saliva conditions and answer a defined clinical question. Under those conditions, plasmonic nanoarrays may support surveillance, risk assessment, and earlier diagnosis without implying that a single platform can diagnose multiple cancers from any convenient fluid.

## Figures and Tables

**Table 1:** Comparison of major biofluids for non-invasive cancer diagnostics.

Biofluid	Main Sampling Advantage	Cancer Context	Representative Biomarkers	Major Analytical Issues
Urine	Large volume; painless repeated collection	Bladder, prostate, kidney/urothelial settings	cfDNA/ctDNA, miRNA, EVs, proteins	Dilution, hematuria, pH/osmolality, urea, salts, storage [14,16]
Saliva	Fully non-invasive; self-collection possible	Oral and head-and-neck cancer	miRNA, transcriptomic markers, proteins, EVs	Mucins, proteases, oral-microbial or food background, and collection/patient-specific variability [15,18]
Other nonblood biofluids	Low procedural burden in selected settings	Indication-specific; outside the main scope	Proteins, nucleic acids, metabolites	Indication-specific evidence and collection procedures vary substantially by fluid [8,12]
Blood	Clinically established liquid-biopsy matrix	Broad systemic oncology applications	ctDNA, CTCs, EVs, proteins	Venipuncture; low tumor fraction in early disease [6,7,13]

**Table 3:** Engineering evidence relevant to reproducibility, scale-up, optical performance, and biointerface translation in representative plasmonic platforms.

Study	Platform/Fabrication	Geometry or Scale	Optical/Analytical Metric	Reproducibility Level	Biointerface/Matrix Evidence	Translational Interpretation
Milenko et al. [69]	UV-NIL; Au-coated C-shaped SERS pattern	Single chip/predefined nanopattern	R6G SERS RSD < 4.3% across five positions; repeated detection-cleaning RSD 8.4%	Within-chip + repeated-use	Dried R6G; no clinical urine/saliva matrix	Useful chip-level uniformity benchmark; not inter-batch or wafer-scale evidence.
Gao et al. 2024 [71]	Au nanoTriangle array-on-gel; active delivery to nanogaps	Nanogap SERS platform; five different batches	SERS batch RSD < 10% (8.1% at 949 cm^−1^; 3.2% at 1016 cm^−1^); protein detection emphasizes hotspot accessibility	Inter-batch	Protein targets in a controlled system; interface designed to reduce nonspecific adsorption	Adds batch-level reproducibility and macromolecule-hotspot-access evidence; not clinical urine/saliva validation.
Zhou et al. 2024 [73]	Defect-tolerant cylindrical SLR array; metal evaporation + NIL	Pillar diameter/height 500 nm; Au 100 nm; period 830 nm; 2 × 2 mm active area	FWHM 5.1 ± 1.1 nm; Q up to 154; simulated refractive-index sensitivities 435 and 733 nm/RIU for the two SLR dips; structural-defect simulations; IgG LOD 609 pg/mL	Inter-batch + within-array: small resonance-wavelength variations among different batches of successively fabricated arrays; spectra consistent at three randomly selected array locations; within-batch processing deviation within 10 nm	Anti-IgG/IgG; spiked artificial serum, with recovered concentrations reported to agree with nominal values at ~99%; no clinical urine/saliva matrix	Experimental NIL example linking defect tolerance, linewidth, and replication; not direct evidence for statistical disorder in self-assembled arrays and not finished-cartridge or clinical urine/saliva validation.
Hwang et al. 2024 [72]	Microbubble-assisted replication of quasi-3D plasmonic nanostructures	4-inch wafer	Wafer-scale replication in <10 min	Wafer-scale manufacturing demonstration	No final functionalized clinical-biofluid assay reported for the manufacturing demonstration	Shows manufacturing scale/throughput; does not establish functionalized assay lot-transfer.
Zhao et al. 2026 [67]	AAO-template-assisted Au/Ag nanoarrays	Particle diameter ~40–80 nm; thickness 20–50 nm; center-to-center distance ~100 nm; n = 1.0–1.6	Geometry-dependent LSPR peak, FWHM, and refractive-index sensitivity	Centimeter-scale/template-assisted fabrication; quantitative inter-batch or wafer-level optical RSD: NR	Refractive-index liquids; no clinical urine/saliva biointerface	Links geometry to optical response; optical sensitivity should not be equated with biochemical LOD.
Feller et al. 2025 [70]	Fluid-interface-assisted colloidal self-assembly and transfer; Au–PNIPAM core–shell microgels, with plasma-treated AuNP lattices	Non-close-packed 2D Bravais lattices; six-position analysis on a cm^2^-scale glass substrate	5.2 ± 0.3 particles/µm^2^ (<10% variation in particle number per unit area); plasmon peak 584 ± 1 nm across positions; experiment–FDTD peak-position/width differences attributed to defects and finite domains; post-treated/overcoated SLR: 617 nm, FWHM 40 nm, Q = 15	Within-substrate positional homogeneity; lot-to-lot calibration transfer: NR	Optical colloidal-lattice study; no biospecific receptor assay or clinical urine/saliva matrix	Direct self-assembled defect/domain-size–linewidth/Q evidence; not evidence of clinical calibration transfer or finished-cartridge reproducibility.

Notes: NR, not reported. Reproducibility levels are kept distinct; within-chip RSD cannot be interpreted as inter-batch or wafer-scale reproducibility. SERS EF values are not tabulated where the calculation/reference condition is not directly comparable; EF definitions and reporting conventions remain non-interchangeable [82]. Quantitative antifouling or anti-contamination retention values are likewise not transferred from adjacent non-plasmonic platforms when they were not measured on the representative plasmonic array and matrix listed here.

## Data Availability

No new data were created or analyzed in this study. Data sharing is not applicable to this article.
